# Mendelian randomization analysis of causal and druggable circulating inflammatory proteins in schizophrenia

**DOI:** 10.3389/fpsyt.2024.1465291

**Published:** 2024-10-31

**Authors:** Hongbao Cao, Li Fu, Dongming Liu, Ancha Baranova, Fuquan Zhang

**Affiliations:** ^1^ School of Systems Biology, George Mason University, Manassas, VA, United States; ^2^ Department of Psychiatry, The Affiliated Brain Hospital of Nanjing Medical University, Nanjing, Jiangsu, China; ^3^ Department of Radiology, Nanjing Drum Tower Hospital, Affiliated Hospital of Medical School, Nanjing University, Nanjing, Jiangsu, China; ^4^ Research Centre for Medical Genetics, Moscow, Russia; ^5^ Institute of Neuropsychiatry, The Affiliated Brain Hospital of Nanjing Medical University, Nanjing, Jiangsu, China

**Keywords:** Mendelian randomization, circulating inflammatory proteins, schizophrenia, gene-drug analysis, GWAS

## Abstract

**Background:**

Schizophrenia (SZ) is a severe mental disorder with complex origins. Observational studies suggested that inflammatory factors may play a role in the pathophysiology of SZ and we aim to investigate the potential genetic connection between them by examining the causal impact of circulating inflammatory proteins on SZ.

**Methods:**

We utilized Mendelian randomization (MR) analysis to assess the causal relationship between circulating inflammatory proteins and SZ and the GWAS summary datasets were sourced from public databases. The SZ dataset comprised 74,776 cases and 101,023 controls, while the summary results for 91 plasma proteins in 14,824 participants were obtained through the Olink Target platform. Moreover, to identify and evaluate potential drug targets, we searched the Drug-Gene Interaction Database (DGIdb).

**Results:**

The results of the MR study confirmed that nine inflammatory proteins had a causal effect on SZ. Among these proteins, IL1A (OR: 0.93), TNFB (OR: 0.94), TNFSF14 (OR: 0.96), and CD40 (OR: 0.95) exhibited protective effects against SZ. Conversely, CCL23 (OR: 1.04), CCL19 (OR: 1.04), 4EBP1 (OR: 1.06), TWEAK (OR: 1.08), and DNER (OR: 1.10) were associated with an increased risk of SZ. The MR-Egger and weighted median methods also supported the direction of these effects. According to the Gene-Drug analysis, LTA, IL1A, CD40, and 4EBP1 can serve as drug targets.

**Conclusions:**

Our study established causal relationships between circulating inflammatory proteins and SZ. It may be beneficial to personalize the treatment of SZ by incorporating inflammation management into the treatment regimen.

## 1 Introduction

Schizophrenia (SZ) is a mental illness characterized by disordered thought form and cognitive deficits, influenced by a combination of genetic and environmental factors. Epidemiological studies indicate that SZ affects about 1% of the worldwide population, posing significant implications for global health (1, 2). SZ not only adversely affects the physical and mental health of individuals but also places a considerable burden on the social healthcare system. The etiology of SZ is multifaceted, involving genetic predisposition, prenatal infections, life pressure, and substance abuse, among other factors (3). Moreover, disturbances in the immune-kynurenine pathway play a significant role in treatment-resistant SZ (1, 4, 5). Although current treatments for SZ include pharmacotherapy and psychotherapy (4, 6), the prognosis is not encouraging due to the severity of the illness, untimely intervention, poor medication adherence, and lack of social support (7).

Inflammatory cytokines are pivotal in regulating neurogenesis, synaptogenesis, and myelination, significantly impacting brain development. However, excessive or prolonged inflammation can disrupt these processes, leading to abnormal brain function and neurodevelopmental disorders (8–10). Dysregulation of inflammatory factors may cause neuronal damage and death, resulting in long-term cognitive and behavioral disorders (11, 12). For example, the involvement of inflammatory factors in the development of optic neuritis and in predicting the progression of multiple sclerosis provide evidence of their significance in neurological diseases (13), while omega-3 polyunsaturated fatty acids show promise in alleviating cytokine-induced neuroinflammation and neurotransmitter dysfunction in SZ (14). Thus, the inflammatory cytokines play a prominent role in both normal and pathological brain development. In terms of SZ, inflammatory cytokines may act by influencing neuroinflammation and neurotransmitter functions.

Some studies have established a close link between inflammatory cytokines and SZ, such as Neutrophil Gelatinase-Associated Lipocalin (NGAL) and Interferon-gamma (IFN-γ), which are related to cognitive dysfunctions in SZ, affecting the severity of the disease (15, 16). Furthermore, elevated levels of pro-inflammatory cytokines are believed to disrupt brain functions and neural signaling pathways (17). For instance, interleukin-6 (IL-6) and tumor necrosis factor-alpha (TNF-α) can undermine normal brain development, leading to neuroinflammation, oxidative stress, and alterations in neurotransmitter signaling, which can be associated with the pathophysiological process of SZ (4). While dysregulation of IL-6 and TNF-α observed in patients with SZ further confirms their involvement in neuroinflammation, reinforcing this connection and potentially serving as targets for therapeutic intervention (18, 19). In addition, IL-1β shows a paradoxical effect, with SZ patients’ microglia displaying reduced responsiveness despite increased inflammation-related gene expression, and elevated IL-1β levels are associated with more severe depressive and negative symptoms, suggesting its potential as a therapeutic target (20, 21). IL-18 is consistently elevated in SZ, with alpha-linolenic acid (ALA) proposed to reduce its levels (22), while IL-12 dysregulation correlates with worse cognitive performance in first-episode psychosis patients [PMID: 37972880] (23). These cytokines highlight the intricate connection between inflammation and SZ. Observational studies have unveiled a connection between inflammatory cytokines and SZ, but the initiation of SZ is affected by diverse factors, encompassing socioeconomic elements and comorbidities. These intricate elements curtail the precision of conventional research methods in exploring the causal impact. Mendelian Randomization (MR) has emerged as a crucial tool for evaluating disease causality due to its distinctive feature of randomly assigning alleles. This characteristic proves effective in circumventing confounding factors and reversing causality (24–26). The strength of MR also lies in its foundation on Genome-Wide Association Study (GWAS) summary data, providing a more flexible and convenient approach to analyzing causality between diseases (27). Therefore, we aimed to analyze the genetic relationship between immunoproteins and SZ with the MR method.

## 2 Methods

### 2.1 Data source

The schizophrenia (SZ) GWAS datasets were originally acquired and analyzed by Trubetskoy et al., and the results were published in Nature in 2022 (28). These data include 74,776 cases and 101,023 controls, making it one of the most comprehensive GWAS studies on SZ to date (28). The populations of the dataset include European (EUR), East Asian (ASN), African American (AA), and Latino (LAT) ancestry, with EUR ancestry (53,386 cases and 77,258 controls) accounting for approximately 70%.

The data for circulating inflammatory proteins were obtained through the Olink Target platform, which analyzed 14,824 individuals of European descent (29). This GWAS study, conducted by Zhao et al. and published in Nature Immunology in 2023, identified 180 protein quantitative trait loci (pQTLs) associated with the levels of 91 plasma proteins (29). Full per-protein GWAS summary statistics are available for download at https://www.phpc.cam.ac.uk/ceu/proteins and the EBI GWAS Catalog (accession numbers GCST90274758 to GCST90274848).

The studies related to the primary data involved in this study have been ethically reviewed, and we only performed secondary analyses of the existing data, so no additional ethical approval was required.

### 2.2 MR analysis

In this study, we used three models in the TwoSampleMR package (version 0.5.6) to infer causality (30). The inverse variance weighted (IVW) method was employed as the primary method, which enhances the precision of results by weighting the estimated values from each instrumental variable (IV) to reduce random error. Meanwhile, the IVW model assumes that all IVs are valid and that their association with the outcome is realized solely through exposure. To ensure the reliability of the results, we also used the Weighted Median (WM) and the MR-Egger model to complement the IVW (31, 32). The WM method assigns greater weight to more precise IVs, providing causal estimates even when some IVs are invalid, and the MR-Egger model can assess and correct for biases caused by invalid IVs.

For sensitivity analysis, we used the intercept of MR-Egger regression to evaluate horizontal pleiotropy (32). A significant non-zero intercept in the MR-Egger regression suggests the presence of horizontal pleiotropy, indicating that some IVs might influence the outcome through pathways other than exposure. Moreover, heterogeneity was assessed by Cochran’s Q test and the I (2) statistic, where a P-value less than 0.05 and an I (2) greater than 0.25 were considered significant.

We selected single nucleotide polymorphisms (SNPs) associated with the phenotype and significant across the genome from the exposure dataset. To incorporate more validated IVs and improve statistical efficacy, we set the threshold at P < 1×10^-5^. We used the 1000 Genomes Project Phase 3 (EUR) as the reference panel and pruned these IVs using an r² threshold of 0.001 within a 10 Mb window. This approach ensured that the SNPs used as IVs were independent, enhancing the accuracy and reliability of the MR results.

### 2.3 Gene-drug interaction analysis

In order to identify and evaluate potential therapeutic targets or mechanisms of drug action, we delved deeply into the existing resources in the Drug-Gene Interaction Database (DGIdb) (33). Interacting drug targets were identified by retrieving genes of inflammatory cytokines that were determined by MR analysis and causally associated with SZ. This process provides a scientific basis for the pharmacological treatment of SZ.

## 3 Results

### 3.1 MR analysis

Our MR analysis identified nine inflammatory proteins with a significant causal relationship with SZ. A total of 32, 31, 34, 21, 39, 16, 20, 29, and 27 IVs were obtained from the exposure datasets for LTA (TNFB, Tumor Necrosis Factor β), CCL23 (C-C Motif Chemokine Ligand 23), TNFSF14 (Tumor Necrosis Factor Superfamily Member 14), IL-1A (Interleukin 1 Alpha), TNFSF12 (TWEAK, Tumor Necrosis Factor-Related Weak Inducer of Apoptosis), 4EBP1 (EIF4EBP1, Eukaryotic Translation Initiation Factor 4E Binding Protein 1), CCL19 (C-C Motif Chemokine Ligand 19), CD40, and DNER (Delta/Notch-Like EGF Repeat-Containing Transmembrane Protein), respectively.

**Figure 1:**
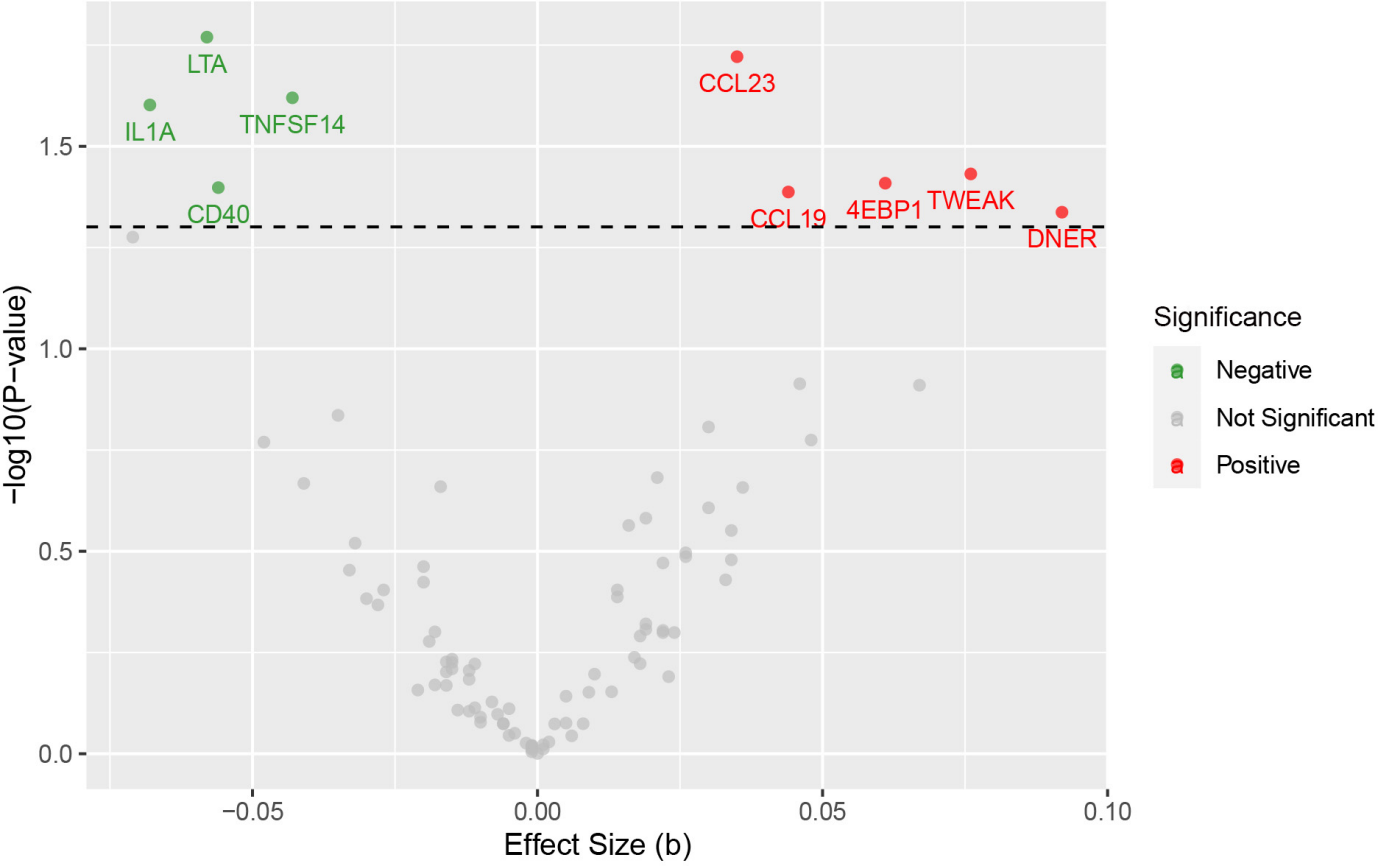
Volcano plot for the causal effects of circulating inflammatory proteins on schizophrenia. The red dots are for positive significant, gray for not significant, and green for negative significant.

**Figure 2:**
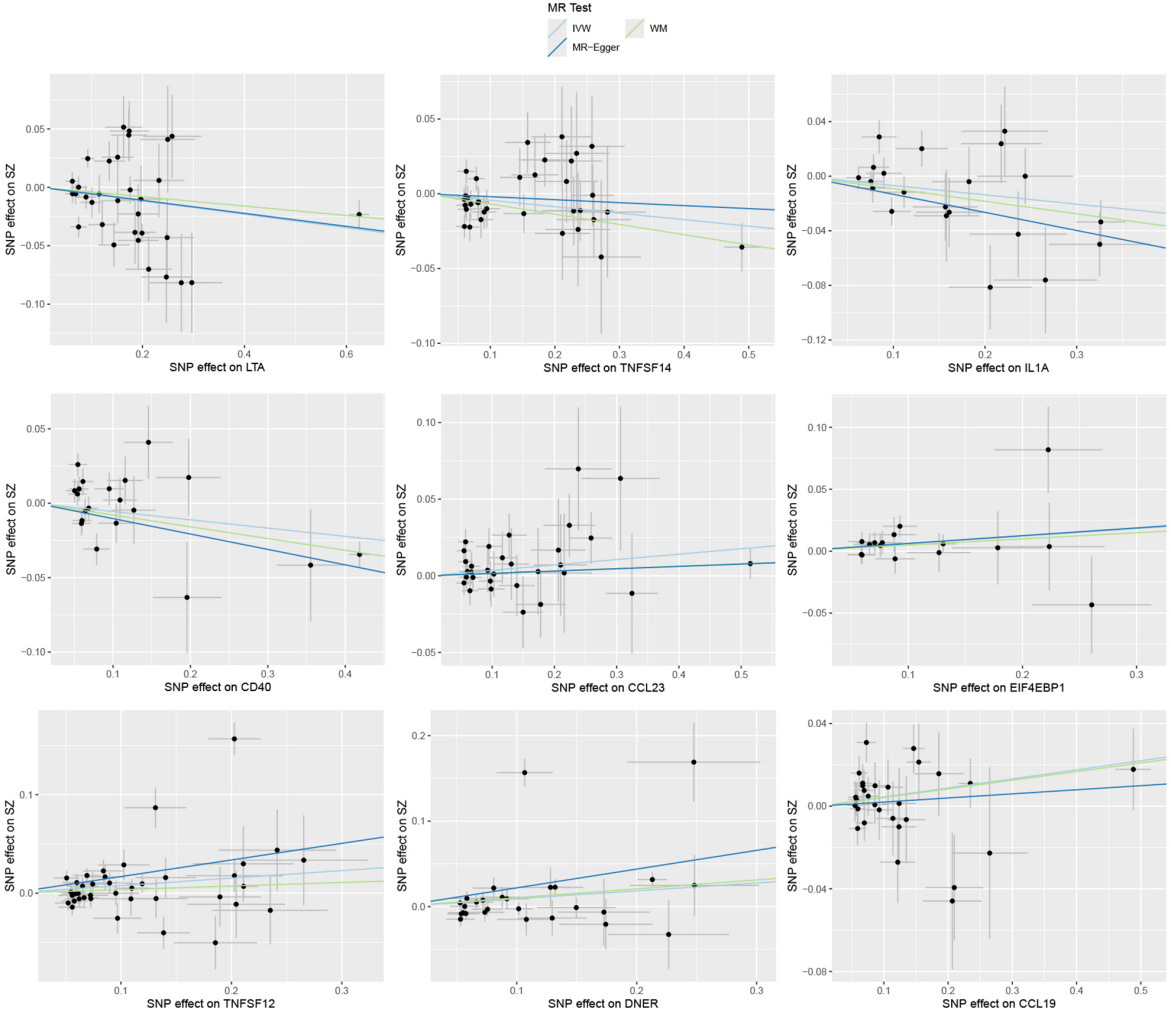
Causal effects of nine circulating inflammatory proteins on schizophrenia. The trait on the x-axis denotes the exposure, the trait on the y-axis denotes the outcomes, and each cross point represents an instrumental variant. The lines denote the effect sizes (b) of the MR analysis.

**Figure 3:**
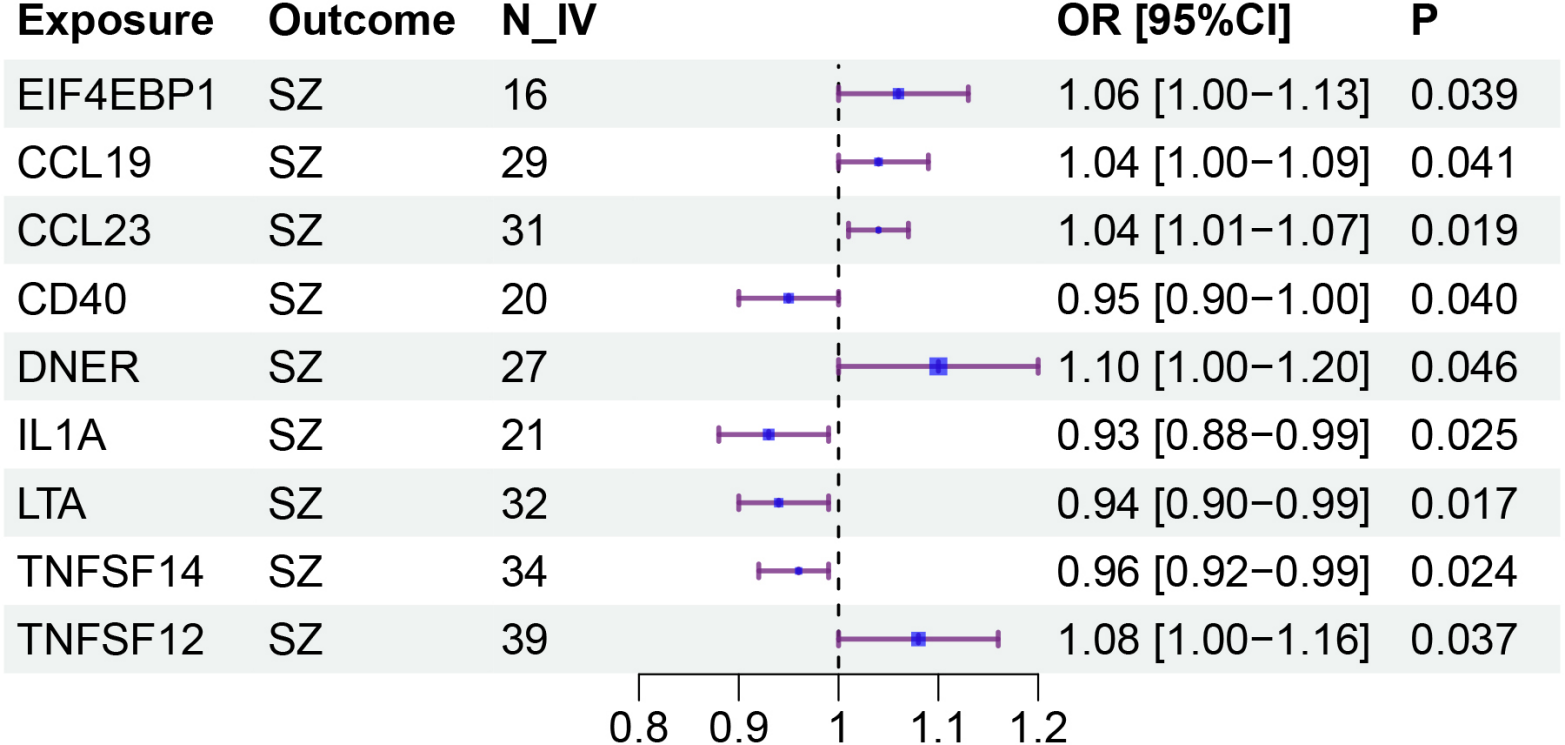
Forestplot for the causal effects of circulating inflammatory proteins on schizophrenia.

Specifically, LTA (OR: 0.94; 95% CI: [0.90-0.99]), IL1A (OR: 0.93; 95% CI: [0.88-0.99]), TNFSF14 (OR: 0.96; 95% CI: [0.92-0.99]), and CD40 (OR: 0.95; 95% CI: [0.90-1.00]) are associated with a reduced risk of SZ. While CCL23 (OR: 1.04; 95% CI: [1.01-1.07]), 4EBP1 (OR: 1.06; 95% CI: [1.00-1.13]), TWEAK (OR: 1.08; 95% CI: [1.00-1.16]), CCL19 (OR: 1.04; 95% CI: [1.00-1.09]), and DNER (OR: 1.10; 95% CI: [1.00-1.20]) may increase the risk of SZ (**Table 1**, **Figures 1**–**3**). Importantly, both MR-Egger and weighted median (WM) methods yielded consistent results in terms of the direction of causal effects. (**Supplementary Tables 1**, **2**)

**Table 1:** Causal effects of circulating inflammatory proteins on schizophrenia.

Exposure	Outcome	N_IV	b (se)	OR [95%CI]	Q_P	I2	P_pleiotropy	P
LTA	SZ	32	-0.058 (0.024)	0.94 [0.90-0.99]	7.64E-08	0.66	0.947	0.017
TNFSF14	SZ	34	-0.043 (0.019)	0.96 [0.92-0.99]	0.234	0.143	0.366	0.024
IL1A	SZ	21	-0.068 (0.030)	0.93 [0.88-0.99]	0.025	0.414	0.212	0.025
CD40	SZ	20	-0.056 (0.027)	0.95 [0.90-1.00]	3.27E-04	0.598	0.079	0.04
CCL23	SZ	31	0.035 (0.015)	1.04 [1.01-1.07]	0.491	-0.016	0.193	0.019
TNFSF12	SZ	39	0.076 (0.037)	1.08 [1.00-1.16]	1.49E-13	0.728	0.202	0.037
EIF4EBP1	SZ	16	0.061 (0.029)	1.06 [1.00-1.13]	0.634	-0.192	0.981	0.039
CCL19	SZ	29	0.044 (0.022)	1.04 [1.00-1.09]	0.223	0.16	0.459	0.041
DNER	SZ	27	0.092 (0.046)	1.10 [1.00-1.20]	1.50E-14	0.789	0.152	0.046

SZ, schizophrenia; OR, odds ratio; CI, confidence interval; N_IV, number of instrumental variables; Q_P, Cochran’s P-value of heterogeneity analysis.

In the sensitivity analysis, the results of the MR-Egger regression indicated no significant horizontal pleiotropy, as evidenced by a P-value greater than 0.05. However, Cochran’s Q test and the I (2) statistic suggested the potential presence of heterogeneity in the MR estimates (P < 0.05).

### 3.2 Gene-drug interaction analysis

After searching the DGIdb, we identified four inflammatory proteins are drug targets, including LTA, IL1A, CD40, and 4EBP1. Specifically, LTA is targeted by Carbamazepine and Abacavir; IL1A is targeted by Hydroxychloroquine, Rilonacept, and Olanzapine; CD40 is targeted by Hydroquinone, Streptozocin, and Fludarabine; and 4EBP1 serves as a target for Paclitaxel, which is used for treating multiple diseases. Among these drugs, only Carbamazepine and Olanzapine are used primarily for treating mental disorders. (**Table 2**)

**Table 2:** The results of Gene-Drug interaction.

Gene	Drug	Regulatory approval	Drug indications	Interaction score
LTA	Carbamazepine	Approved	For treatment of bipolar disorder	0.67
LTA	Abacavir	Approved	NA	1.41
IL1A	Hydroxychloroquine	Approved	Antirheumatic agent	1.87
IL1A	Rilonacept	Approved	Antiinflammatory agent, DMARD	20.61
IL1A	Olanzapine	Approved	Antipsychotic agent	0.31
CD40	Hydroquinone	Approved	NA	0.66
CD40	Streptozocin	Approved	NA	
CD40	Fludarabine	Approved	Antineoplastic agent	0.59
4EBP1	Paclitaxel	Approved	For treatment of peripheral arterial disease (PAD); antineoplastic agent; anti-inflammatory agent, DMARD	1.27

DMARD, Disease-Modifying Antirheumatic Drugs; NA, Not applicable.

## 4 Discussion

While numerous studies have described the connection between inflammation factors and SZ (4, 14, 15), research on their genetic relationship is relatively scarce. Our study bridges this gap, and the results of MR indicated that circulating inflammatory proteins contribute to a genetic susceptibility to SZ.

In the study, we identified four inflammatory factors—LTA, IL1A, TNFSF14, and CD40—that demonstrated a negative correlation with SZ, suggesting a protective function against the SZ. Conversely, CCL23, CCL19, 4EBP1, TWEAK, and DNER exhibited a positive correlation, implying an increased risk of SZ. These findings provide a crucial genetic perspective for gaining a deeper understanding of the pathophysiological mechanisms underlying SZ.

The CD40 and its ligand CD40L are transmembrane proteins belonging to the tumor necrosis factor receptor (TNFR) superfamily. CD40, acting as a cell surface receptor, protects SZ by regulating immune responses and reducing neuroinflammation. Research has indicated that prenatal immune activation can lead to SZ by disrupting pathways associated with CD40, such as the CD200-CD200R and CX3CL1-CX3CR1 pathways (34), and the observed positive correlation between brain-derived neurotrophic factor (BDNF) and CD40L in SZ patients suggests a protective effect on neurons (35). Additionally, the elevated levels of soluble CD40L found in both bipolar disorder and SZ indicate the change in the inflammatory system (36), which suggests that activating the CD40 signaling pathway could trigger the release of anti-inflammatory cytokines and neuroprotective factors, potentially slowing the development and progression of SZ.

Lymphotoxin A (LTA), also called TNFB, is a key cytokine closely associated with SZ, primarily produced by lymphocytes (37). It plays a significant role in brain functions such as neurodevelopment and response to neural injury (38), and the variations in the LTA gene have been linked to an increased risk of developing SZ, particularly in the Korean population (39). Moreover, LTA is believed to be linked to the neurodevelopmental hypothesis of SZ, as it can prevent prenatal infections and mitigate the neurotoxic effects of glutamate on neurons. LTA also has the ability to regulate the synthesis and secretion of key cytokines (like IL-6 and TNF-α) and neurotrophic factors, as well as influencing synaptic plasticity. These functions not only modulate neuronal activities but may also play a positive role in alleviating the symptoms of SZ (40).

Additionally, the expression of TNFB mRNA in white and gray matter regions of the brain that are sensitive to excitotoxins, particularly in key areas like the hippocampus, demonstrates a direct neuroprotective effect on neurons (40, 41). This effect is crucial for maintaining the health and balance of the nervous system, especially in response to neural stress and injury. TNFB also stimulates the release of the neurotransmitter nitric oxide (NO), optimally regulating microglia and exerting a neuroprotective effect (40, 42). This could be significant in mitigating the development of SZ. Moreover, carbamazepine as an antiepileptic drug is able to effectively improve the affective symptoms of schizophrenia by affecting neurotransmitters (43). According to the results of our gene-drug interaction analysis, it may work through LTA as a target.

TNFSF14 is a member of the tumor necrosis factor (TNF) superfamily (44). As a key immunoregulatory factor, TNFSF14 not only acts as a pro-inflammatory agent enhancing immune responses but can also produce different effects by binding to various receptors. When TNFSF14 binds to the LTB receptor, it can activate T cells and promote their proliferation, working in conjunction with other co-stimulatory molecules to elicit immune responses. Whereas, TNFSF14 can act as an inhibitor when it binds to the DcR3 receptor and modulates immune responses (45). Moreover, our findings suggest that TNFSF14 has a protective effect against SZ. Thus, the role of TNFSF14 in regulating the neuroinflammation associated with SZ deserves further exploration.

IL1A is an important pro-inflammatory cytokine, not only eliciting inflammatory responses via IL-1R1 but also functioning as a damage-associated molecular pattern (DAMP) (46). Although some genetic studies have suggested that IL1A is associated with susceptibility to SZ, others have provided differing insights and indicated that its dysfunction may be related to the pathogenesis of schizophrenia (47). Our research showed that there is a negative correlation between IL1A and SZ, potentially reducing the risk of SZ. One possible mechanism for this association is that IL1A enhances the body’s immune defense by promoting the infiltration of immune cells such as neutrophils (46). Additionally, IL1A may affect the development of SZ by influencing neurotransmitter pathways such as glutamate and GABA (48).

Olanzapine, targeting IL1A and serving as an atypical antipsychotic, is widely used in the treatment of SZ. It improves negative symptoms and cognition of SZ by regulating dopamine release and physiologic brain activity (49). Hydroxychloroquine may serve as an effective adjunctive medication for treating SZ by exerting anti-inflammatory effects on pro-inflammatory factors (50, 51). Therefore, exploring the protective mechanism of IL1A in SZ is a direction with significant value.

Chemokines play a significant role in the central nervous system, mediating the migration and transport of leukocytes and participating in the regulation of neuroinflammation, neurotransmission, and neuroendocrine functions (52, 53). CCL19 is a member of the CC family of immune chemokines and enhances the immune responses of CD8+ cells and macrophages (52). It also binds to the chemokine receptor CCR7, functioning in tissue immunity and inflammatory responses (54). Elevated levels of chemokines such as CCL19 have been found in neuroinflammatory diseases and psychiatric disorders such as SZ, suggesting a potential link to it (53, 55, 56). Thus, dysregulation of CCL19 levels may interfere with immune responses and neuroinflammation, potentially leading to the pathophysiological processes of SZ.

CCL23, also known as Chemokine β8-1 (Ckβ8-1), Myeloid Progenitor Inhibitory Factor 1 (MPIF-1), and Macrophage Inflammatory Protein 3 (MIP-3), is a chemokine closely associated with inflammatory responses (57, 58). This factor is primarily secreted by macrophages and contributes to immune reactions through mechanisms such as promoting the migration of immune cells and stimulating the production of pro-inflammatory factors and adhesion molecules (57–59). Elevated levels of CCL23 in cerebrospinal fluid and serum, serving as inflammatory markers, indicate its significant role in neuroinflammation and immune dysregulation (54, 57). Additionally, the involvement of CCL23 in neuroinflammation associated with neurodegenerative diseases such as Alzheimer’s Disease (AD) and Parkinson’s Disease (PD) also provides corresponding evidence (58, 60). Therefore, by enhancing neuroinflammatory levels, CCL23 may impact cellular functions and brain structure, subsequently increasing the risk of SZ (61, 62). Further investigation is required to elucidate the specific mechanisms by which CCL23 may contribute to the pathogenesis and progression of SZ.

4EBP1 is a key protein involved in protein synthesis and regulation of neuronal function (63). The main mechanism of 4EBP1 is to reduce protein synthesis by inhibiting translation initiation and reducing eIF4E activity (64). A study suggested that treatment with MK-801 in experiments may alleviate the symptoms of SZ by promoting protein translation. In this context, 4EBP1 inhibition of translation may alter the expression of key synaptic proteins and neural signaling pathways involved in the pathogenesis of SZ, and increase the risk of the disease (65).

TNFSF12, also known as TWEAK, is a transmembrane protein of the TNF superfamily ligand group and is expressed in brain tissue. TWEAK can disrupt the permeability of the blood-brain barrier by promoting cytokine secretion, leading to brain tissue damage (66). And as a cytokine involved in inflammation and immune responses, TWEAK may play a key role in the development of SZ by affecting neuroinflammation and synaptic dysfunction (67). Changes in TWEAK levels observed in the serum and cerebrospinal fluid of SZ patients further suggest its association with the pathogenesis of the disease. The study has indicated that lower TWEAK levels found in male patients may be related to neurodevelopment, while higher serum levels of TWEAK might be involved in inflammatory processes (67, 68). However, further research is needed to determine the specific nature and significance of this association.

Delta/Notch-like epidermal growth factor (EGF)-related receptor (DNER) is specifically expressed in the central nervous system and functions in its development (69). Primarily located within dendritic cells, DNER acts as a receptor or ligand to alter synaptic states, thereby mediating protein-protein interactions (69). DNER also functions as a ligand in the Notch signaling pathway, binding to Notch receptors and activating signal transduction to regulate interactions between neurons and glial cells (70).

Additionally, DNER contributes to inhibiting the proliferation of neural progenitor cells and promoting the maturation and differentiation of glial cells (71). Different types of glial cells, through mechanisms such as immune regulation, synaptic pruning, and elimination, can cause brain dysfunction and neurotransmitter dysregulation (72). Although current research supports the result that DNER can elevate the risk of developing SZ, more investigations are necessary to comprehensively understand the specific mechanisms through which DNER influences SZ.

In addition to acting independently, the inflammatory factor CD40 can collaborate synergistically with 4EBP1, TNFSF14, TWEAK, and DNER to regulate the recruitment and activation of immune cells, thereby influencing the progression of SZ (73, 74). The interaction between CD40 and TNFSF14 can modulate the signaling pathways of inflammatory responses (75). As part of the TNF ligand family, CD40 also interacts with TWEAK by affecting its expression and the activated Fn14-TWEAK system can inhibit CD40 signaling (76). Furthermore, regulation of CD40 affects DNER expression and signal pathways with consequences for neuronal differentiation and synaptic function (77).

CD40 also promotes the translation of its mRNA by isolating 4EBP1 (78). It induces the phosphorylation of 4EBP1 to segregate it from eIF4E, enhancing translation initiation and leading to the activation of immune responses and increased protein synthesis (79). And dysregulation of 4EBP1 could disrupt CD40 signaling, impairing immune function and increasing susceptibility to diseases.

## 5 Limitations

Despite the significant findings of our study, several limitations should be noted. First, the summary datasets used in the MR analysis primarily include individuals of European ancestry, which may limit the generalizability of the results to other populations. Future studies with more diverse datasets are needed to validate these findings across different ethnic groups.

Second, while MR analysis is a powerful tool for inferring causality, it is reliant on the assumption that the selected instrumental variables (pQTLs) are valid and influence the outcome solely through the exposure (inflammatory proteins). Although we used complementary methods such as MR-Egger to account for pleiotropy, the possibility of residual pleiotropy or confounding cannot be entirely ruled out.

Third, heterogeneity was observed in some of the MR estimates, suggesting potential variability in the effect of certain proteins on schizophrenia. This heterogeneity may arise from differences in the genetic instruments or underlying biology, and further research is needed to explore these inconsistencies.

Lastly, the functional mechanisms through which the identified proteins influence schizophrenia remain unclear. While we identified potential drug targets, experimental studies are required to validate these findings and explore the clinical relevance of targeting these proteins in schizophrenia treatment.

## 6 Conclusions

In conclusion, our study establishes causal relationships between circulating inflammatory proteins and schizophrenia, identifying both protective and risk-associated factors. Using Mendelian Randomization analysis, we found nine inflammatory proteins, including IL-1A and TNF-α, that contribute to the genetic susceptibility of schizophrenia.

These findings enhance our understanding of the role of inflammation in schizophrenia and highlight potential therapeutic targets, such as LTA and CD40, for future drug development. Incorporating inflammation management into treatment regimens may also improve outcomes for patients.

Further research is needed to validate these associations across diverse populations and to explore the mechanisms through which these proteins influence schizophrenia. Overall, our results underscore the importance of inflammation in schizophrenia and suggest new avenues for therapeutic strategies.

## Data Availability

The original contributions presented in the study are included in the article/**Supplementary Material**. Further inquiries can be directed to the corresponding author.
